# High-Velocity Penetrating Abdominal Injury Secondary to a Motorized Wire Brush in the Workplace

**DOI:** 10.7759/cureus.28214

**Published:** 2022-08-20

**Authors:** Nicholas Bahl, Monica Sciturro, David Lowery

**Affiliations:** 1 Surgery, Regional Medical Center Bayonet Point, Hudson, USA; 2 Medicine, Dr. Kiran C. Patel College of Osteopathic Medicine, Nova Southeastern University, Clearwater, USA; 3 General Surgery, Largo Medical Center, Largo, USA

**Keywords:** penetrating trauma, removal of foreign body, occupational injuries, undifferentiated abdominal pain, penetrating abdominal injury

## Abstract

Penetrating injuries are one of the most common types of workplace accidents. The majority of these injury patterns are due to non-missile type, low-velocity sharp objects. We present an uncommon presentation of subacute abdominal pain secondary to non-missile type, high-velocity workplace injury. It is important to keep a wide differential in mind when evaluating patients with lingering abdominal pain without an obvious cause. Additionally, collecting the patient's employment history, occupational exposures, and job tasks are important when a workplace injury is suspected. We present the case of a 59-year-old male with abdominal pain for five weeks. Outpatient CT scan of the abdomen/pelvis demonstrated a metallic foreign body within the abdominal cavity. The patient underwent laparoscopic removal of the intra-abdominal foreign body while utilizing fluoroscopy. He recovered without sequelae and he was free of abdominal pain at six months postoperatively. Overall, it is important to maintain a wide differential when evaluating atypical abdominal pain.

## Introduction

Penetrating injuries are one of the leading types of workplace accidents that present to the emergency department. In 2013, Ada et al. found that 35.6% of patients admitted for occupational injuries presented with a penetrating injury, with a majority of these coming from the metal and metal-based products industry [1]. The severity of these injuries is dependent on both the characteristics of the organ affected and those of the penetrating object, i.e. sharpness and size.

Anatomically, the small bowel is the most common organ injured in penetrating abdominal trauma, accounting for 49%-60% of all injuries, due to it occupying a large volume of the peritoneal cavity. It is also attached to the mesentery, making it highly mobile and vulnerable [2]. In this case report, the vulnerability of the small bowel mesentery was evident and its mobility added an extra challenge to the metallic wire removal. Additionally, penetrating abdominal injuries in the workplace are mostly caused by low velocity and non-missile type sharp objects [3]. However, in this case, the penetrating injury resulted from a missile type, high velocity 5 cm x 0.1 cm wire.

## Case presentation

**Figure 1:**
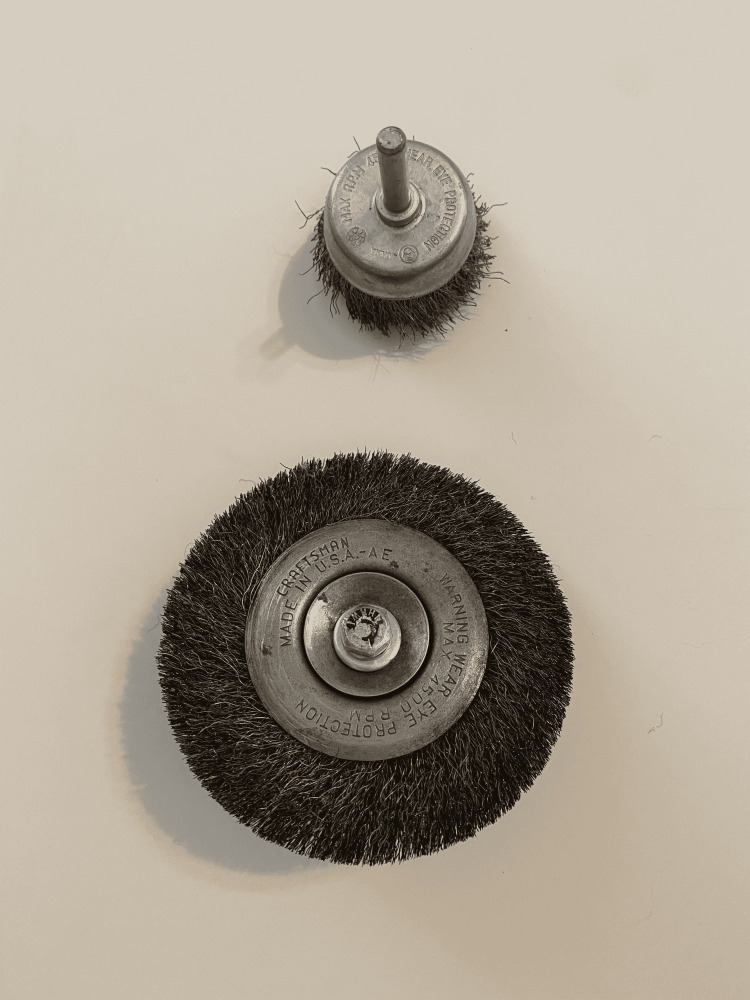
Example of the type of wire brush used by the patient

Our patient was an otherwise healthy 59-year-old male who presented to the general surgery clinic with almost five weeks of mainly right-sided abdominal pain. He stated he was cleaning with a motorized wire brush while wearing a shirt on his upper body (Figure 1). Since that time he has had intermittent sharp, stabbing pain in his right abdomen. He was afebrile and denied any night sweats, bowel changes, nausea, or episodes of emesis. A focused abdominal exam elicited tenderness to palpation to the right hemiabdomen. There was no evidence of rebound, guarding, or generalized peritonitis. In addition, there were no obvious changes to the abdominal wall skin, and no signs of puncture, wound, or erythema. A CT scan of his abdomen/pelvis demonstrated a likely wire metallic object in his right hemiabdomen. His white blood cell count was 7.4 K/uL, with no left shift.

**Figure 2:**
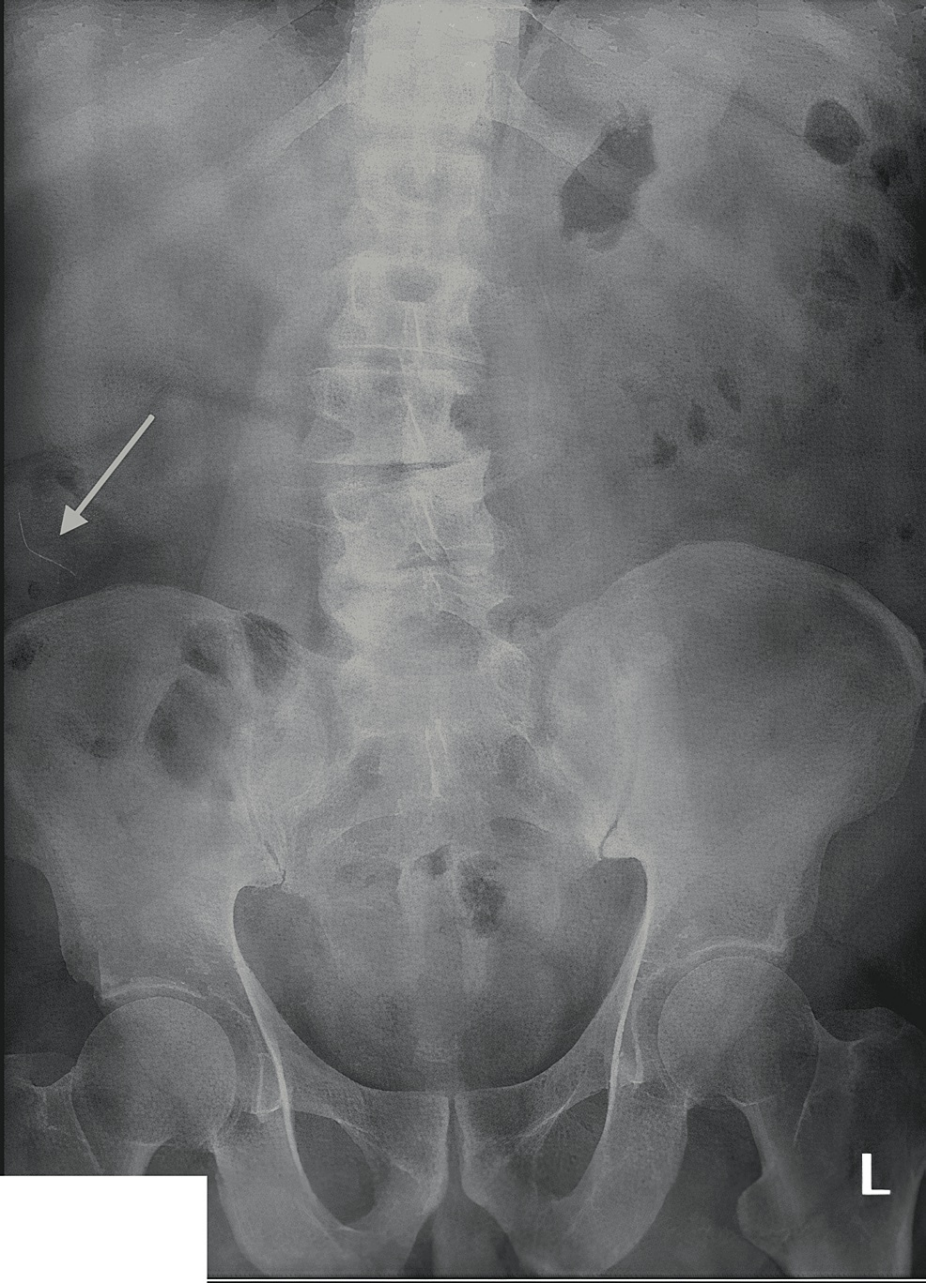
Pre-operative abdominal radiograph demonstrating a curvilinear density within the right lower quadrant (white arrow noting radiopaque density)

**Figure 3:**
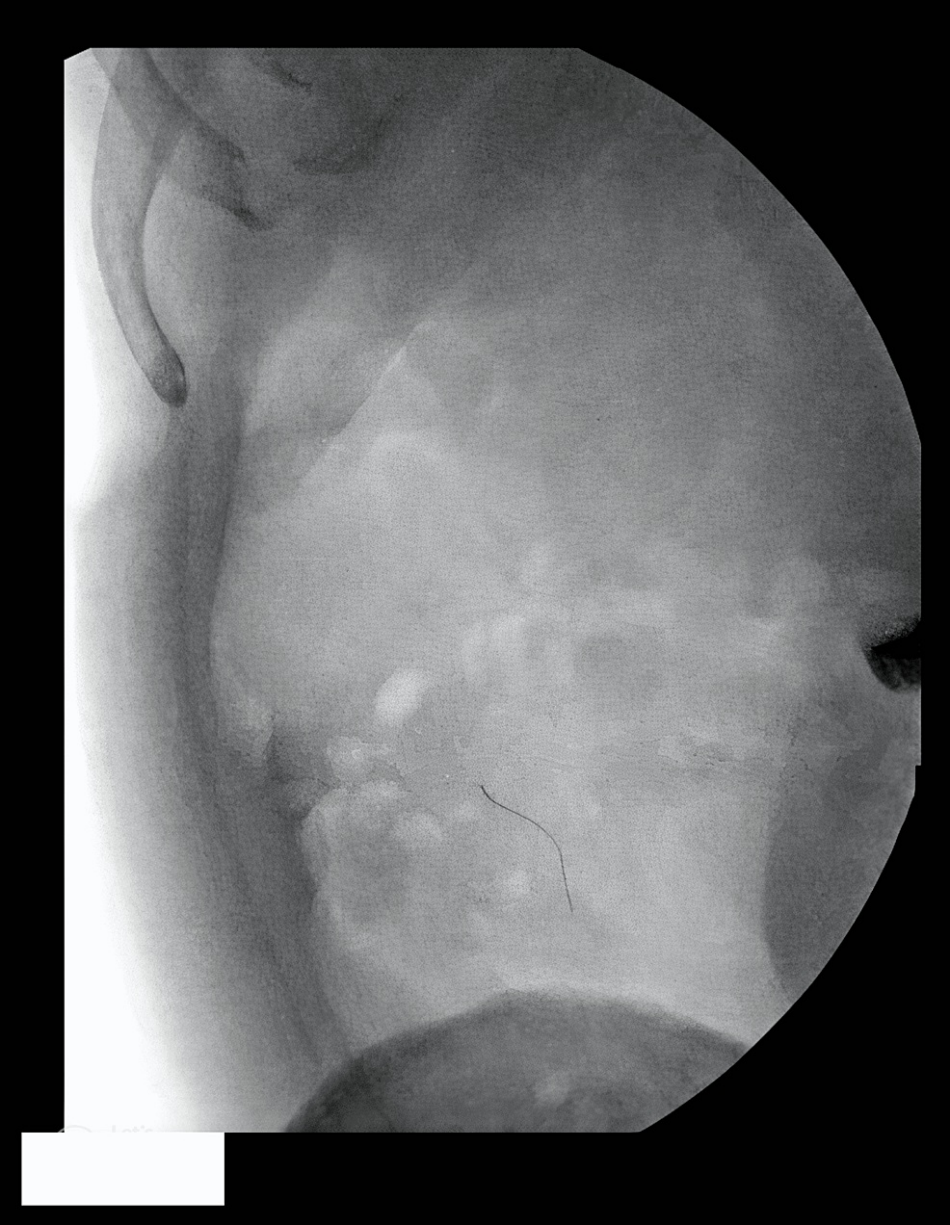
Intraoperative fluoroscopy demonstrating right lower quadrant radiopaque foreign body

**Figure 4:**
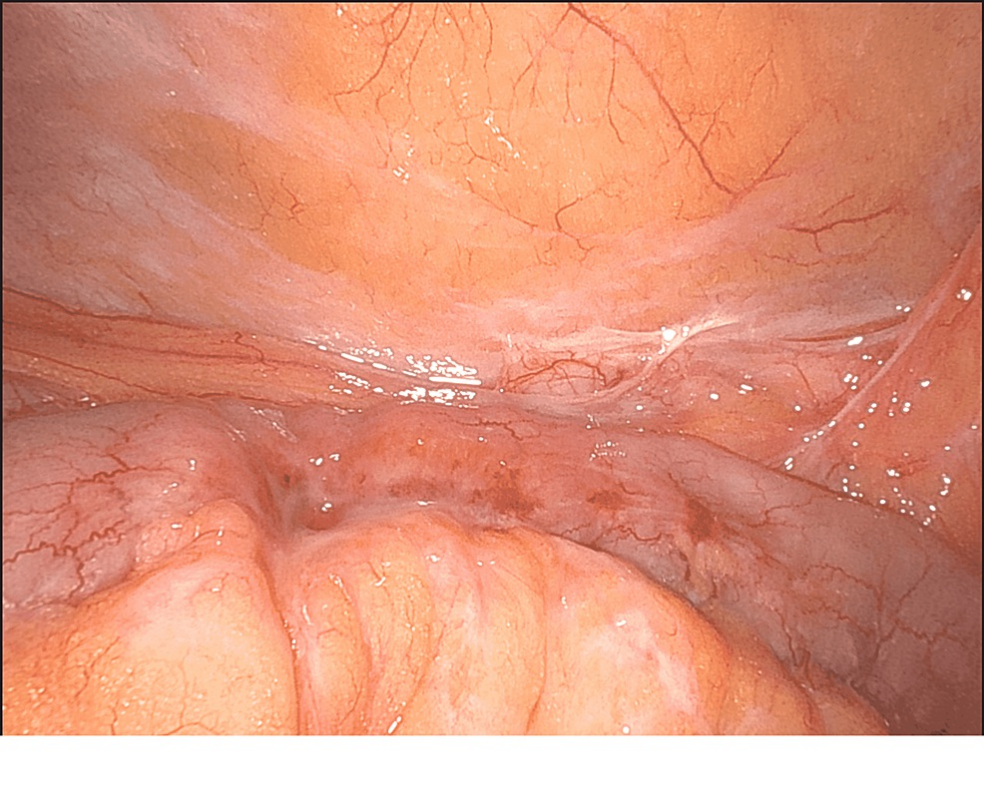
Inflammatory reaction and serosal injury to the small intestine secondary to the wire

**Figure 5:**
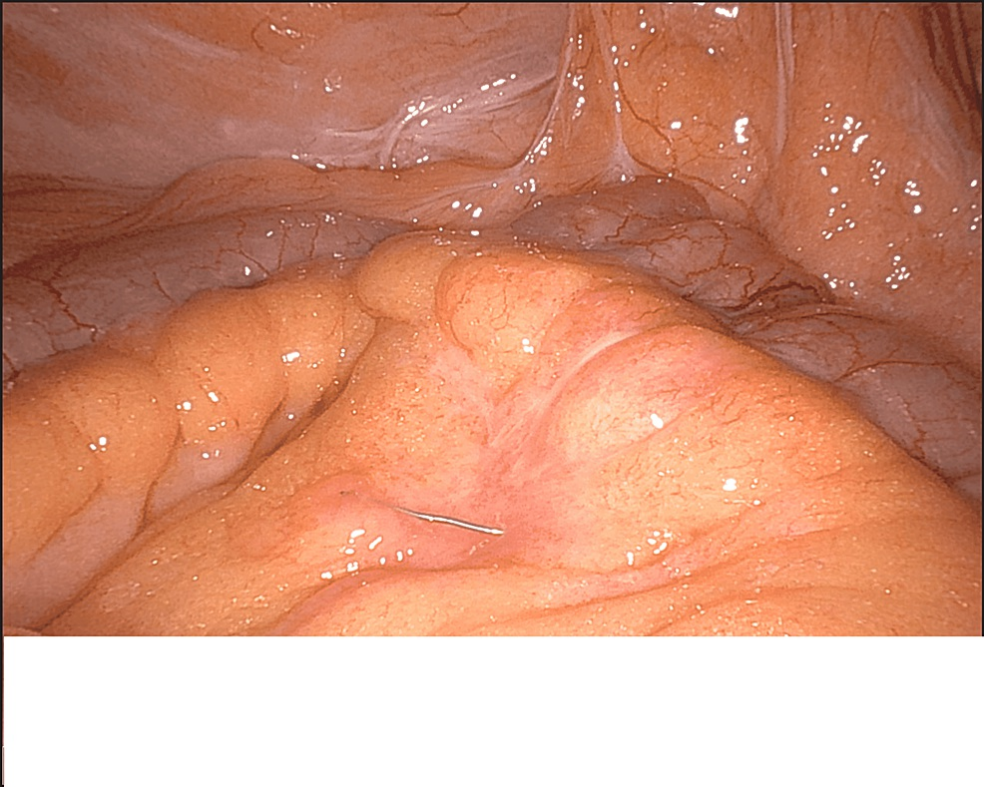
Wire embedded in the small intestine mesentery

**Figure 6:**
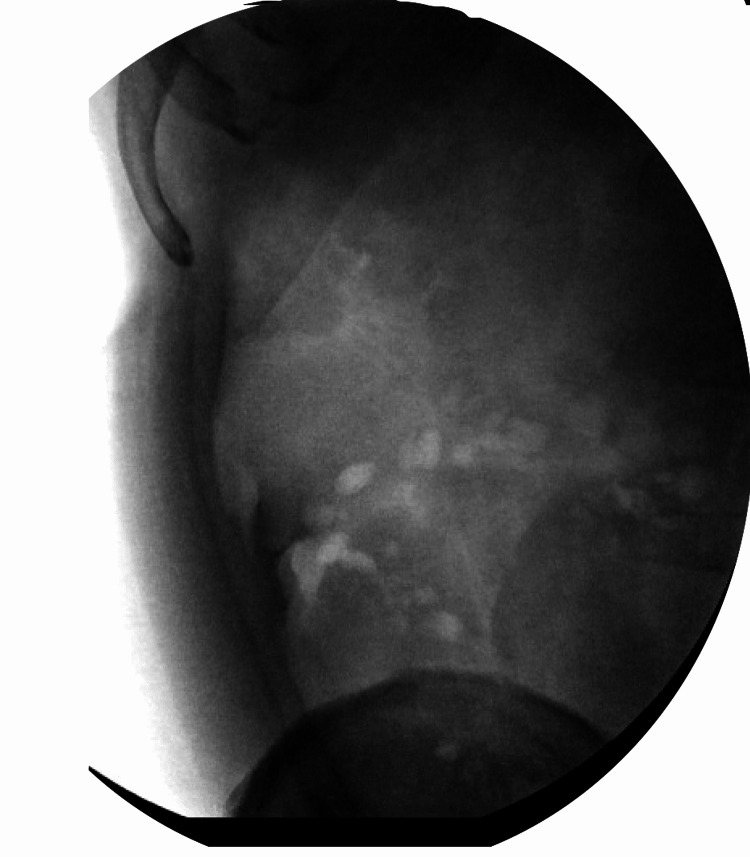
Post removal of foreign body fluoroscopic image of the abdomen; note the absence of radiopaque foreign body

**Figure 7:**
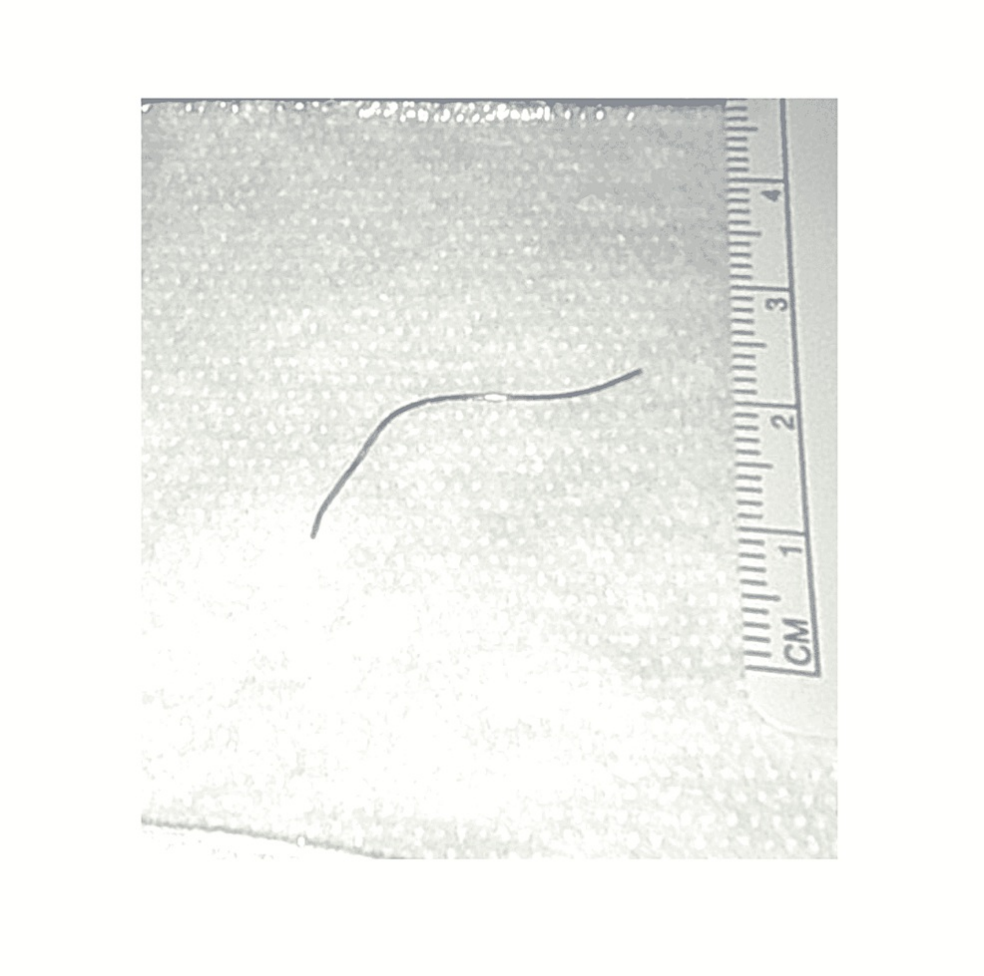
Wire after removal

After the metallic object was found on CT, diagnostic laparoscopy was discussed and the patient agreed to proceed. On the day of surgery, the patient underwent a pre-operative abdominal radiograph which demonstrated the likely wire metallic object in his right hemiabdomen (Figure 2). During the diagnostic laparoscopy, two laparoscopic bowel graspers were utilized to reflect the small intestine medially and fluoroscopy was utilized to identify the object within the abdomen (Figure 3). The foreign body was appreciated and adhered to the small intestine mesentery and a Maryland dissector was utilized to grasp and remove the foreign body from the mesentery (Figure 4). Additionally, there were some abrasions of the small intestine serosa, likely secondary to the foreign object, without evidence of leak or perforation (Figure 5). These appeared to be only a couple of mm in diameter. Post removal fluoroscopic image was obtained which demonstrated the absence of the metallic foreign body (Figure 6). The remainder of the abdomen was without abnormalities. The liver was smooth without any gross nodularity, the appendix was non-dilated, and both inguinal canals were without any gross hernia defect. The foreign object was sent for pathology which demonstrated a 3 cm x 0.1 cm gray/silver metallic wire (Figure 7). 

The patient tolerated the procedure and was discharged. At six months follow-up, the patient was doing well without any recurrence of his abdominal pain

## Discussion

Common agents causing penetrating wounds in the workplace include pneumatic or spring air guns, needles, and sharp machinery [4]. Metal spikes, wooden stakes, and vehicular and industrial instruments have been reported as well. Few reports of penetrating projectiles from hand tools have also been reported. One similar case presentation was published discussing a penetrating abdominal injury caused by a metallic projectile in the workplace but unfortunately, it was fatal [3]. As seen in both our patient presentation and the additional case reported, individuals who work in the metal industry are at risk for penetrating injuries as grinders can be dangerous, and shattered or detached pieces can act as high-velocity projectiles.

Occupational injuries continue to significantly affect the United States workforce with 2.5 million non-fatal injuries reported in 2020 [5]. In a study of trauma patients from 1996-2010, 7% of trauma patients presented with an injury that occurred in the setting of the workplace [6]. A large portion of these injuries can be attributed to a lack of safety precautions or device malfunctions. Injuries to the upper extremities, trunk, and lower extremities far outnumber the injuries to other body systems. Of these, 23% of injuries were to the trunk, with abdominal injuries accounting for 25% of trunk injuries [7]. This case highlights the potential need for additional safety precautions when working with power tools and with wire brush attachments, considering both workplace and penetrating trauma accidents are increasing with one study showing an incidence of 4.17/year up to 8.53/year [6].

## Conclusions

Penetrating abdominal injuries continue to be one of the leading causes of workplace injuries that present to the emergency department. For patients presenting from undifferentiated abdominal pain and working in at-risk occupational environments, there should be a high index of suspicion for underlying traumatic injury. In this case, we described a patient presenting with a penetrating injury to the small bowel mesentery secondary to a missile type, high-velocity metallic wire from a metal wire brush. This case emphasizes the importance of a detailed history and broad differential in cases of undifferentiated abdominal pain and illustrates the need for increased workplace safety to protect from possible device malfunctions.
